# Multifunctional Pomegranate Peel Microparticles with Health-Promoting Effects for the Sustainable Development of Novel Nutraceuticals and Pharmaceuticals

**DOI:** 10.3390/plants13020281

**Published:** 2024-01-18

**Authors:** Milica Radan, Nada Ćujić Nikolić, Snežana Kuzmanović Nedeljković, Zorana Mutavski, Nemanja Krgović, Tatjana Stević, Smilja Marković, Aleksandra Jovanović, Jelena Živković, Katarina Šavikin

**Affiliations:** 1 Institute for Medicinal Plants Research “Dr. Josif Pančić”, Tadeuša Košćuška 1, 11000 Belgrade, Serbiatstevic@mocbilja.rs (T.S.);; 2 Institute of Technical Sciences of SASA, Knez Mihailova 35/IV, 11000 Belgrade, Serbia; smilja.markovic@itn.sanu.ac.rs; 3 Institute for the Application of Nuclear Energy INEP, University of Belgrade, Banatska 31b, 11080 Belgrade, Serbia; acancarevic@tmf.bg.ac.rs

**Keywords:** pomegranate peel, microencapsulation, spray drying, antioxidant, hypoglycemic, antimicrobial activity, molecular docking, in vitro release

## Abstract

Recovering the bioactive components from pomegranate peel (PP) in the fruit-processing industry has attracted great attention in terms of minimizing the waste burden, as well as providing a new source of a multitude of functional compounds. The present study aimed to develop a feasible microencapsulation process of PP extract by using pectin and a pectin/2-hydroxypropyl-*β*-cyclodextrin (HP-*β*-CD) blend as coating materials. Microsized powders obtained by a spray drying technique were examined in terms of technological characteristics, exhibiting high powder yield and desirable moisture content, flowability, and cohesive properties. Assuming that the interactions with the used biopolymers occur on the surface hydrophobic domain, their presence significantly improved the thermal stability of the microencapsulated powders up to 200 °C. The health-promoting effects of PP have been associated with its high content in ellagitannins, particularly punicalagin. The obtained PP powders exhibited strong antioxidant and hypoglycemic potential, while an antimicrobial assay revealed their potent activity against Gram-positive bacteria. Additionally, an in vitro release study suggested that the used biopolymers can modify the release of target bioactive compounds, thus establishing a basis for developing an oral-controlled release system. Altogether, biowaste valorization from PP by the production of effective multifunctional microsized powders represents a sustainable way to obtain novel nutraceuticals and/or pharmaceuticals.

## 1. Introduction

The problem of residual biomass waste from the food-processing industry has become the focus of intense global scrutiny due to the significant nutrient and energy resources that can meet the needs of the world’s growing population [1]. The nearly 59 million tons of food waste generated annually raises the issue of its disposal as well as the socioeconomic and environmental consequences [2]. For that reason, the adoption of sustainable practices for further waste valorization is a priority area in the EU’s Circular Economy Action Plan.

Pomegranate (*Punica granatum* L., Punicaceae) is the edible fruit of a small leafy tree or shrub native to Southwest Asia and extensively cultivated in the Mediterranean, South Africa, and the United States [3]. The production and consumption of pomegranate keeps increasing owing to the fruit’s taste and nutrition, which is commonly consumed fresh or processed into juice. Nevertheless, the peel of the pomegranate fruit, which accounts for approximately 50% of the total weight, is usually discarded as waste [4]. The generation of 669 kg of waste for processing one ton of fresh pomegranate fruit can impact ecosystems and agricultural lands [5]. Therefore, turning pomegranate peel (PP), which accounts for 78% of the generated pomegranate waste, into a value-added product is in line with the principles of sustainability and food-waste reduction. As a rich source of polyphenols, including phenolic acids, flavonoids, and tannins, PP was reported to have a broad range of biological activities and health-promoting benefits, such as antioxidant, antimicrobial, anti-inflammatory, antimutagenic, and apoptotic [6,7,8,9,10,11]. The most abundant bioactive compounds are ellagitannins, including punicalagin and punicalin, gallic acid, and ellagic acid, which were demonstrated to have various protective effects on the human body and have a great potential to ameliorate chronic metabolic diseases, including cardiovascular diseases, diabetes, and obesity [12,13,14]. In this regard, PP could be used as a promising raw material for the production of extracts with multifunctional physiological effects that can provide auxiliary strategies for the treatment of related human diseases.

The key stages in acquiring high-quality extracts are the selection of an appropriate solvent and extraction technique. Methanol, acetone, and ethanol are commonly used organic solvents for the extraction of naturally derived compounds from plant materials. However, the ethanol–water mixture is generally recognised as a safe, easy-to-handle, nontoxic and environmentally acceptable solvent [15]. In comparison to the advanced extraction techniques which require high pressure, temperature, microwave, or ultrasonication, percolation is a conventional extraction method that still stands as one of the most frequently used in the industry sector, as it is relatively simple, convenient, and inexpensive. The advantage of this method is that it consumes less power and can be easily scaled up to facilitate large production of PP extracts at an industrial level.

Polyphenolic compounds derived from natural products possess certain drawbacks associated with their poor stability and bioavailability, which challenge the pharmaceutical product’s formulation and development [16]. Due to the presence of multiple hydroxyl groups, they are susceptible to degradative reactions like epimerization and oxidation which consequently lead to a decrease in their stability and, thus, their shelf life [17]. In this regard, the implementation of the microencapsulation processes using biocompatible carriers represents a promising approach for the preservation of valuable bioactive compounds. Spray drying is one of the most frequently used techniques for plant polyphenol encapsulation into a biopolymer matrix to obtain high-quality microsized powders and maintain their functional and biological characteristics [16,18]. It represents a simple, efficient, high-capacity, and cost-effective conventional method, which converts liquid extract into a powder in a stream of heated air. Among other parameters, the microencapsulation efficiency highly depends on the selected coating material which protects the core medium against external conditions. Most of the coating materials currently applied in the microencapsulation processes mainly refer to polysaccharides and proteins. Cyclodextrins (CDs) are cyclic oligosaccharides that have gained substantial attention in the field of pharmaceutics and technology, for the preservation of biomolecules from environmental influences. They are characterised by a truncated cone-shaped structure, hydrophobic inside and hydrophilic on the outside, which enables them to act as cage macromolecules [19]. Among others, 2-hydroxypropyl-*β*-cyclodextrin (HP-*β*-CD) has an advantage in human use due to its higher solubility and lower toxicity, with a significant effect on the increased photostability, solubility, and bioavailability, as well as the scavenging capability, of phenolic compounds [20]. In order to produce the desired therapeutic effects, bioactive components need to be released from their carriers. The incorporation of CDs into an oral dosage form is a well-known strategy to control the slow release of pharmacologically active components [21]. Moreover, CDs could be used as permeation enhancers in topical dosage forms, increasing the permeability of compounds with poor water solubility by making them available at the biological membrane surface [21]. Combining barrier properties of different naturally occurring carriers or their chemical modifiers in the microencapsulation process could be the key to the successful preservation of phenolic compounds. The outstanding properties of pectin, including its biocompatibility, biodegradability, and low toxicity, have established its extensive use as a coating biopolymer. Chemically, pectin is a polysaccharide that mainly consists of *α*-D-galacturonic acid residues linked by partially methoxylated glycosidic bonds. Moreover, it has several therapeutic benefits, including anti-inflammatory, hypoglycemic, immunoregulatory, antibacterial, antioxidant, and antitumor activities, which could potentiate the biological activity of PP extracts [22]. 

Several previous studies have reported the microencapsulation of phenolic compounds from PP with various coating materials and methods [23,24,25,26]. However, there is a lack of knowledge concerning the development of multifunctional microsized powders of PP extract with pectin and HP-*β*-CD together via the spray drying method. Considering the great therapeutic potential of PP extract, this study aims to establish a feasible process of microencapsulation using these two biocompatible carriers in order to obtain preserved systems with highly potent bioactive components.

## 2. Results and Discussion

In recent years, polyphenols have attracted a great deal of attention due to their prominent role in the control or prevention of oxidation processes and associated diseases. PP, as a rich potential byproduct resource, offers several functional and nutraceutical qualities owing to its bioactive ingredients. Herein, the formulated spray-dried powders of PP extract were characterised based on their physicochemical properties, in addition to the biological evaluation of antioxidant, hypoglycemic (α-amylase, α-glucosidase), and antimicrobial activities. To further disclose the molecular mechanism of hypoglycemic potential, a molecular docking analysis of the predominant bioactive compounds from PP extract and α-amylase and α-glucosidase enzymes was performed. In addition, the kinetics of polyphenol release from PP spray-dried powders was assessed in in vitro simulated gastric fluid (SGF) and simulated intestinal fluid (SIF), which could benefit the future development of preparations with a control release profile.

### 2.1. Technological and Physicochemical Characterization of the Microparticles

#### 2.1.1. Powder Yield (PY)

Spray drying represents a convenient and well-known method for obtaining dried powders by transforming the liquid extract into a powder. The PY in the spray drying process can be influenced by various factors, including the properties of the liquid extracts, added carriers, process conditions (pressure, inlet and outlet temperature, liquid feed, and airflow rate), and spray dryer performances [27]. The process can be considered successful if the PY is greater than 50%, which ensures the profitability of employing this technique for obtaining dried extracts (powders) [28]. Herein, the drying process was carried out with the pure extract and with the addition of pectin and a pectin–HP-*β*-CD blend. The obtained values of PY varied from 78.23% to 82.21%, with no statistically significant difference between the samples, implying an efficacious drying process (Table 1). The highest yield was observed for the pure PP extract (82.21%). Likewise, the addition of pectin and a pectin–HP-*β*-CD blend evidenced high PYs (near 80%). The obtained values of PY are significantly higher compared to the results reported by Endo et al. [29], who produced microparticles of PP extracts by spray drying using alginate and chitosan as carriers (40% and 41%, respectively).

#### 2.1.2. Moisture Content (MC)

The MC in spray-dried powders represents a powder characteristic with great importance. Excess moisture in powders can lead to product spoilage, reduced shelf life, and compromised structural integrity, as well as influence the flowability of bulk solids [30]. The MC of the prepared PP microparticles varied between 2.43% and 3.38%. A slightly higher value was observed for the powder produced with pectin–HP-*β*-CD (3.38%) in comparison to the powders produced without a carrier and with pectin only (2.51% and 2.43%, respectively). HP-*β*-CD is a cyclic oligosaccharide that is chemically modified to improve its solubility and other properties. Its hydroxypropyl groups and cyclic structure contribute to its ability to efficiently encapsulate and interact with guest molecules as well as water. As a result, HP-*β*-CD is often more directly hygroscopic compared to natural polysaccharides such as pectin [31]. However, according to the *European Pharmacopoeia* (Ph. Eur.) 11th Edition, all the obtained powders meet the criteria of dried extracts since their MC values were below 5% [32]. Hadree et al. [33] analysed pomegranate powder enriched with the phenolic extract of PP by spray drying with an MC between 7.84% and 11.39%, which was two-to-three-fold higher than in the powders obtained in this research.

#### 2.1.3. Bulk Density

Each pharmaceutical form has its challenges in terms of storage, further processing, and transportation. The bulk density is one of the important powder parameters that determines, among others, the quality of the final product. A high bulk density indicates that the powder is more uniform (with fewer air gaps between the particles), which can increase powder stability [26]. Its values for the obtained PP powders ranged from 0.23 to 0.32 g/mL (Table 1). The powder containing pectin and HP-*β*-CD (C) had a bulk density that was not significantly different from the pure extract powder (A), indicating that the addition of this combination of carriers did not negatively affect the quality of the powder. On the other hand, a slightly lower value was observed for the PP powder with pectin (B). The same trend was observed for the tapped density.

The Carr index (CI) and Hausner ratio (HR) are parameters frequently used in pharmacy to evaluate the flowability and cohesiveness of powders, respectively, both providing complementary information on different aspects of the flow powder properties. The CI evaluates compressibility, while the HR reflects packability and cohesion [34]. A CI value higher than 25 can be considered an indicator of poor powder flowability, while a value less than 15 indicates good flowability; meanwhile, powders with an HR higher than 1.25 can be considered to flow poorly [35]. According to Shah et al. [34], powder C demonstrated good flowability, meanwhile, powder A possessed poor flowability. According to the results, the use of pectin and an HP-*β*-CD mixture significantly improved the characteristics of the obtained PP powders. Therefore, HP-*β*-CD increases the bulk density of powders during the microencapsulation process by forming inclusion complexes with the active ingredients. The molecular structure of HP-*β*-CD and its ability to reduce interparticle porosity contributes to the overall increase in bulk density during the encapsulation process [36]. 

#### 2.1.4. Rehydration

The rehydration time of spray-dried powders can vary depending on several factors, including the specific powder composition, particle size, and rehydration conditions. The rehydration process involves the absorption of water by the powder particles, which can be influenced by factors such as surface area, porosity, and solubility of the powder [37]. The results presented in Table 1 showed that the rehydration time of the studied samples ranged from 21.12 to 108.8 s, revealing that the carrier’s addition increased the time required for dissolving the powders. A longer rehydration time with carrier addition can extend the release of bioactive compounds and achieve controlled and sustained bioactive release [38].

#### 2.1.5. Particle Size Distribution

**Figure 1:**
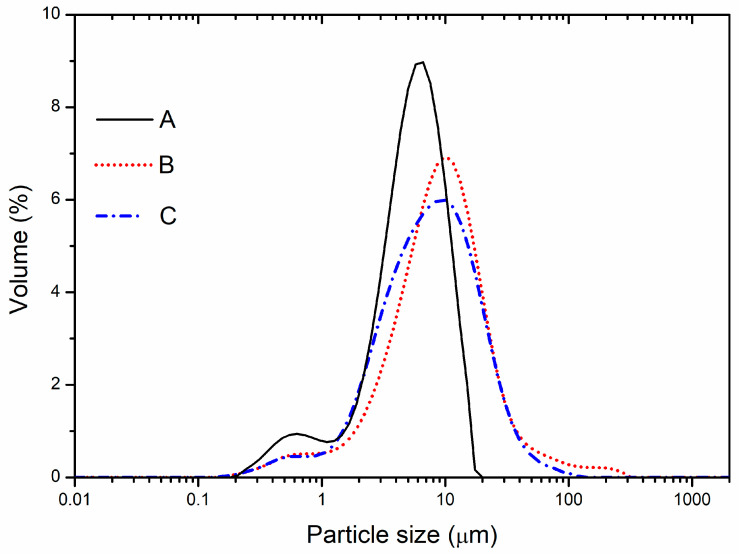
Particle size distribution of the obtained spray-dried powders of pure pomegranate peel (PP) extract (A), PP extract with 10% pectin (B), and PP extract with a mixture of 10% pectin and 5% 2-hydroxypropyl-*β*-cyclodextrin (HP-*β*-CD) (C).

Spray drying as a microencapsulation technique can produce particles of different sizes depending on the parameters of the drying process, the used biopolymers, and their concentration, as well as the microencapsulated bioactive compounds [39]. The particle size of the obtained PP micropowders was ascertained using the laser diffraction method (DLS), and the obtained results demonstrated three different peak modal distributions, bimodal for pure spray-dried PP extract and almost unimodal for the microencapsulates (Figure 1). The DLS analysis indicated that the PP powders could be classified as microparticles (Table 2), with the mean particle size ranging from 5.05 µm for spray-dried PP extract to 8.43 µm for micropowder produced with pectin [40]. It could be observed that the used biopolymers increased the particle size diameter of microencapsulates, especially those with pectin, which is in agreement with our previous study [18]. Jovanović et al. and Ćujić Nikolić et al. [41,42] confirmed analogous observations. Meanwhile, Tonon et al. [43] related this phenomenon to the increased viscosity of the feed solutions. Particles prepared with carrier’s blend exhibited a d_50_ lower than the values obtained for simple pectin. Specifically, sample A was characterised as highly uniform, with a low polydispersity index (PDI) value and superior particle size distribution, followed by sample C. The slightly higher PDI for sample C is probably a consequence of the pectin and HP-*β*-CD tendency to agglomerate [44]. The increasing particle size and PDI values are associated with the properties of the pectin as a wall material [41,45]. Pasrja et al. [46] also demonstrated the tendency (property) of HP-*β*-CD to self-agglomerate, resulting in particles with heterogeneous distribution. The dried extracts showed a high uniformity of particles, with a small mean particle diameter, confirming that spray drying performances are suitable for this type of phytoextract. The temperature during the spray drying process is a major factor influencing particle size, and with a relatively low employed inlet temperature, low moisture content and low particle size diameter were achieved.

#### 2.1.6. Fourier-Transform Infrared (FTIR) Spectroscopy

**Figure 2:**
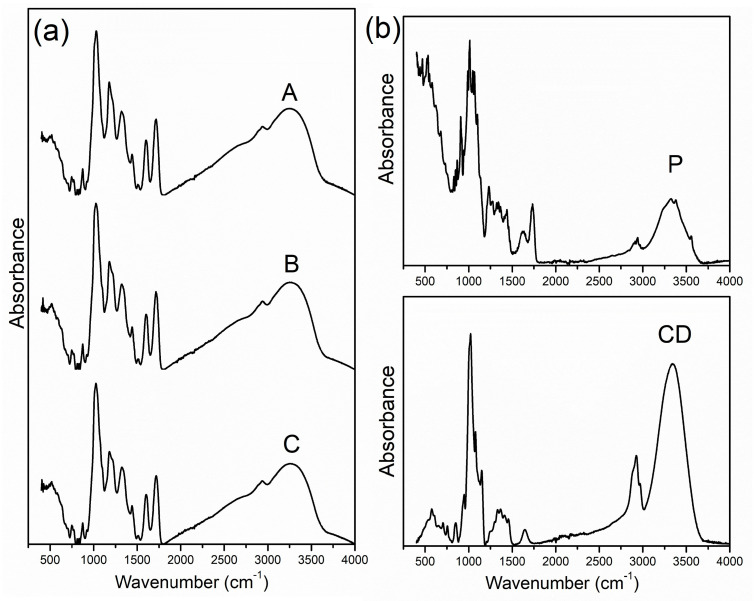
FTIR analysis of the obtained (**a**) spray-dried powders of pure pomegranate peel (PP) extract (A), PP extract with 10% pectin (B), and PP extract with a mixture of 10% pectin and 5% 2-hydroxypropyl-*β*-cyclodextrin (HP-*β*-CD) (C), and (**b**) used biopolymers, pectin (P), and HP-*β*-CD (CD).

FTIR spectroscopy is a powerful technique that has been extensively employed for the chemical and structural characterization of various plant extracts and microencapsulates, detecting functional groups and characterizing bonding information [19,42]. Herein, it was performed to prove the chemical compatibility of the PP extract with the examined carriers. The FTIR spectra of PP spray-dried extract, as well as microparticles obtained with pectin and the pectin–HP-*β*-CD blend are shown in Figure 2.

The characteristic bands of the PP powders that appeared in the fingerprint region are in line with the previously published work revealing the presence of a wide variety of compounds [47,48]. The obtained spectra showed a wide peak between 3200 and 3400 cm^−1^, which could be assigned to the O-H stretching vibrations, while the peak detected at 2937 cm^−1^ indicated the stretching vibration of the alkyl group [47]. The fingerprint region between 1500 and 2000 cm^−1^ possesses two sharp peaks characteristic of the FTIR spectrum of PP extract. The midintense peak at 1714 cm^−1^ can be related to the carbonyl group (C=O), which could be associated with the presence of aldehydes, ketones, and carboxylic acids, while another peak at 1601 cm^−1^ is associated with the C=C stretching vibrations of the unsaturated compounds (alkenes) [47]. The band at ~1395 cm^−1^ can be related to carboxylates, while the peak at ~1350 cm^−1^ can be associated with the OH bending of the alcohol and phenol groups [49]. The peaks at ~1250 cm^−1^ and ~1040 cm^−1^ can be attributed to the stretching vibration absorption peaks of C–O and C–OH of polyphenols in the PP extract [50,51]. 

The FTIR spectra of the used biopolymers are also presented in Figure 2 and show characteristic signals of saccharides attributed to the –OH stretching vibrations at ~3300 cm^−1^, C–H stretching vibration at ~2900 cm^−1^, and O–H bending vibration around 1600 cm^−1^, as well as the C–O vibration at ~1100 cm^−1^ [19,52,53]. According to the literature, specific intensity bands below 900 cm^−1^ in the “fingerprint” region of pectin are mainly ascribed to vibrations of the C–O–C bridges, which is typical for polysaccharides [53].

The obtained spectra of the PP microcapsules did not show any significant difference when compared to the carrier-free powder, suggesting that chemical reactions did not occur during the spray drying process. Since the microencapsulation with pectin and HP-*β*-CD did not affect the characteristic spectrum of PP, and specific fingerprint regions of carriers were overlayed by the spectrum of the extract, it could be concluded that the complexation process occurred on the surface hydrophobic domain [54].

#### 2.1.7. Differential Scanning Calorimetry (DSC)

**Figure 3:**
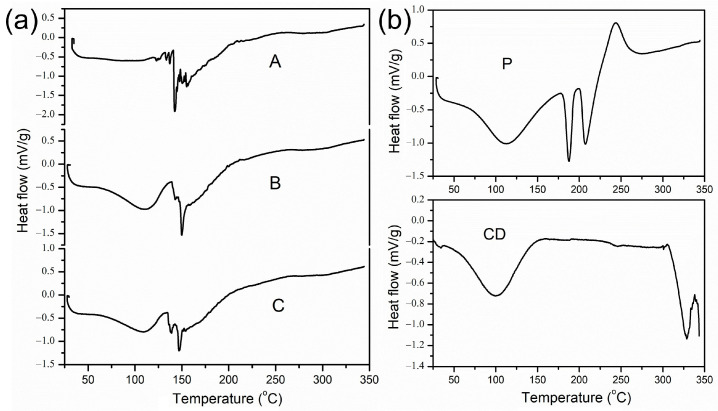
DSC diagrams of the obtained (**a**) spray-dried powders of pure pomegranate peel (PP) extract (A), PP extract with 10% pectin (B), and PP extract with a mixture of 10% pectin and 5% 2-hydroxypropyl-*β*-cyclodextrin (HP-*β*-CD) (C), and (**b**) used biopolymers pectin (P) and HP-*β*-CD (CD).

The thermal behaviour of the samples in the solid state (the pure spray-dried PP extract and the microencapsulates with added biopolymers) was determined by DSC analysis and shown in Table 3 and Figure 3. The PP spray-dried extract and carrier blends showed the characteristic degradation processes of all three components, the extract and the two carriers (Table 3, Figure 3). From the initial temperature (20 °C) to 50 and 60 °C, no thermal activity of the powders was observed, indicating good thermal stability, which is important for pharmaceutical and food-storage processes. The decomposition curves for all analysed samples started around 60 °C (probably due to the water content and low alcohol residues in the extract), and the main weight loss finished at around 200 °C. This first stage of weight loss could be related to structural water loss during evaporation [55]. The PP powder without a carrier exhibited the peak maximum of the initial stage at around 100 °C, while the addition of biopolymers, pectin, and CD drove the temperatures of degradation upwards. The pure PP spray-dried powder demonstrated a multistep degradation pattern, with the most relevant enthalpy change of T_2_ at around 142 °C. The exhibition of several small degradation peaks (Figure 3) was a general characteristic of pure extract and was also revealed with chokeberry and bilberry spray-dried powders [18]. The addition of the carriers began to show two clearly defined temperature changes, and DSC analysis was used to confirm the formation of the inclusion complexes in the solid state. The disappearance of several thermal peaks of the PP extract after inclusion in the carriers and their reduction may indicate successful microencapsulation [56]. The enhancement of PP polyphenols could be explained by the formation of complexes with pectin and CD. It is known that the formation of complexes with CDs can increase thermal stability due to the interaction with the inner cavity [57,58]. The DSC curves of both used biopolymers demonstrated high degradation temperatures, confirming that they influenced the stabilization of microencapsulates compared to the pure spray-dried powder. The highest enthalpy change was observed in the first stage of sample B, indicating higher stability against increased temperature, especially compared to the respective A sample. Furthermore, there was a statistically significant shift in the main degradation stage to 150 °C for sample B, with a final degradation temperature of 198.8 °C. For samples B and C, the first thermal changes were observed at around 100 °C to 110 °C, which were similar to the temperature changes of pectin and HP-*β*-CD. Meanwhile, changes around 150 °C almost certainly originate from the carbohydrate’s degradation of biopolymers [59]. The thermal composition of microencapsulates was superimposed to the higher temperature degradation, which was similar to the results of Tonon et al. [43] of acai spray-dried powders and reduced several temperature changes to two separate ones. Peaks around 300 °C indicated melting and thermal decomposition of HP-*β*-CD [44]. The lack of exothermic peaks in pure spray-dried PP extract and microencapsulates indicated good thermal stability, and according to the results, the microencapsulates verified higher thermal stability due to the synergy between the PP extract and the wall materials. The fact that pectin and HP-*β*-CD may be incorporated into the final products makes these microencapsulates very attractive from the point of view of bioactive compounds’ safekeeping.

### 2.2. Phytochemical Analysis of the Microparticles

#### 2.2.1. Total Phenolic Content (TPC)

Phenolic compounds are the main secondary metabolites of PP extract, whose content in the prepared powders varied from 373.15 to 427.88 mg GAE/g DW (Table 4). The highest yield was observed in the carrier-free powder (A), while slightly lower values were measured in the microencapsulates with pectin (B) and pectin–HP-*β*-CD (C). As expected, the addition of a carrier in the proportion of 10% pectin (B) or 10% pectin and 5% HP-*β*-CD (C) consequently led to a decrease in the phenolic content due to the dilution effect. Different experimental factors, such as the employed extraction method, solvent type, and concentration, can affect the content of total phenolics. Živković et al. [60] have performed ultrasound-assisted extraction (UAE) of PP, with the TPC varying between 118.01 and 190.94 mg GAE/g DW. Moreover, Habchi et al. [61] have revealed that the UAE was more efficient than maceration, with up to 189.45 mg GAE/g of the total polyphenols extracted from ethanolic PP extracts.

#### 2.2.2. HPLC Analysis of Individual Compounds

Pomegranate peel is a well-known source of several structural forms of tannins, with ellagitannins as the most dominant phytochemicals. In the prepared PP microparticles, punicalagin was found to be the main ellagitannin constituent, with content ranging from 112.09 to 126.82 mg/g DW (Table 4). The highest content of this bioactive compound was detected in the carrier-free powder, while slightly lower values were observed in the powders with pectin (B) and pectin–HP-*β*-CD (C), without any statistically significant differences between them (Table 4). As in the case of total phenolics, encapsulation with carriers reduced the amount of PP extract used for spray drying, which consequently resulted in a lower content of bioactives compared to the pure extract. In addition to punicalagin, punicalin was also identified as a representative of this group, with similar content in all three samples that varied between 32.72 and 37.16 mg/g DW (Table 4). Other substances that are present in PP are hydroxybenzoic derivatives, gallic acid, and ellagic acid. The content of ellagic acid in the prepared microsized powders ranged from 9.62 to 11.21 mg/g DW, while gallic acid was quantified between 4.18 and 5.03 mg/g DW (Table 4). In both cases, a slightly higher content was observed in the pure spray-dried PP powder, while comparable results were achieved using pectin or a pectin–HP-*β*-CD blend as carriers. Other authors have also shown the dominance of these compounds in peel extracts, whose concentrations vary among different pomegranate cultivars [62,63].

### 2.3. Biological Evaluation of the Microparticles

#### 2.3.1. DPPH Assay

Plant polyphenols have drawn increasing attention due to their potent antioxidant capacity, which allows them to act as reducing agents, hydrogen donors, or singlet oxygen quenchers. The dried extracts of PP were examined for their antioxidant capacities by measuring radical-scavenging activity in the 2,2-diphenyl-1-picrylhydrazyl (DPPH) assay (Table 5). Phenolic compounds possess an ideal chemical structure for free-radical scavenging activity due to the presence of multiple phenolic hydroxyl groups that are prone to donate an electron or a hydrogen atom to a free radical, as well as an extended conjugated aromatic system that can delocalise an unpaired electron [64]. As presented in Table 5, the highest antioxidant activity was measured for PP powder without carrier (A), while B and C exhibited slightly lower potency due to the dilution effect, i.e., lower content of phenolic antioxidants. Among the microencapsulated extracts, the sample with pectin–HP-*β*-CD mixture (C) showed higher scavenging capacity compared to the sample with pectin alone (B). Although a lower IC_50_ value was observed for standard ascorbic acid (4.45 ± 0.05 µg/mL), it is still comparable to those obtained for the prepared PP microparticles. Previous in vitro studies have shown that the high antioxidant capacity of PP extract is mainly attributed to hydrolysable tannins, specifically ellagitannins, which showed even higher antioxidant activity than the edible part of the fruit [65].

Similar antioxidant activities were found in the peel extracts of pomegranate cultivars from Morocco (12.49 ± 0.60 μg/mL), and those reported by Dadwal et al. (6.12 ± 1.05 μg/mL) [66,67]. Yang et al. [23] evaluated the antioxidant activity of PP microencapsulates prepared with spray drying using two wall-forming components, maltodextrin and pectin. They suggested that using a combination of both carriers was a better approach than using a single one, revealing a better protecting and preserving potential of the antioxidant properties of phenolic compounds during microencapsulation [23]. Consistent with some previous findings, the obtained results imply that the presence of pectin and HP-*β*-CD could synergistically potentiate the antioxidant activity of bioactive compounds due to their unique physicochemical characteristics [18,68]. The formation of supramolecular structures with CDs, which are stabilised via noncovalent bonds, provided an additional benefit by protecting bioactives against rapid oxidation by free radicals [69]. The literature also shows that HP-*β*-CD could act as a secondary antioxidant and, therefore, increase the system’s natural antioxidant capacity [70,71].

#### 2.3.2. In Vitro Hypoglycemic Activity

Diabetes is a heterogeneous metabolic disorder with a rapidly increasing global prevalence characterised by hyperglycemia in both postprandial and fasting states. Slowing down glucose release and absorption plays a key role in the management of diabetes mellitus type 2. Inhibition of carbohydrate-digesting enzymes, such as α-amylase and α-glucosidase, is an effective therapeutic approach to suppress the digestion of nonabsorbable polysaccharides or disaccharides to absorbable monosaccharides [72]. In this study, the hypoglycemic potential of the pure PP powder and PP powders prepared with different carriers by a spray drying technique was investigated through an in vitro analysis of α-amylase and α-glucosidase inhibitory activities. The findings of the present study showed that all the studied PP samples strongly inhibited α-glucosidase and, to a lesser extent, α-amylase, as previously reported by Colantuono et al. [73]. The obtained activities of the PP microparticles against α-glucosidase (Table 5) were also significantly higher than that of acarbose (IC_50_: 156.64 ± 16.63 µg/mL). The carrier-free sample and the sample with a pectin addition showed the best α-glucosidase inhibitory activities with the same IC_50_ values of 0.25 µg/mL (Table 5). Slightly lower activity was observed for the extract microencapsulated with pectin and HP-*β*-CD, which could be attributed to the increase in total carrier amount. Considering the results obtained in the α-amylase inhibitory assay, the extract with pectin was observed to have the highest activity, followed by the extract without the carrier addition and the extract with the pectin–HP-*β*-CD blend, without any statistically significant difference between the obtained IC_50_ values (Table 5). The inhibitory effects of PP powders obtained for α-amylase were lower compared to the standard acarbose (IC_50_: 2.06 ± 0.48 mg/mL) [74]. The higher potency of the sample with pectin could be described by the fact that pectic polysaccharides can inhibit the amylase enzyme activity as documented in several previous studies [75,76]. In particular, Espinal-Ruiz et al. [75] revealed that the inhibitory effect of pectic polysaccharides on the enzyme activity appears to be due to a noncompetitive enzyme–pectin interaction. The potential of PP extracts to exhibit hypoglycemic effects has been highlighted in numerous in vitro and in vivo experiments [13,72,77,78]. Bellesia et al. [79] identified punicalagin, punicalin, and ellagic acid as the main inhibitors of the α-glucosidase enzyme. In addition, Mirab et al. [74] showed that the hypoglycemic potential of PP extracts could be also mainly related to the α-amylase inhibitory effects of ellagic acid.

#### 2.3.3. Antimicrobial Assay

The emergence of growing antimicrobial resistance (AMR) has become a global threat, requiring the search for new therapeutic solutions for treating bacterial and fungal infections. Plant polyphenols proved to have antibacterial and antifungal potential; thus, they may be the solution for the increasing resistance [80]. While the antimicrobial potential of polyphenols may be useful in the pharmaceutical and healthcare sectors, there is also a growing interest in using these naturally derived products as preservatives in food and cosmetic products, as they can decompose easily and therefore cause no toxicity to human health and the environment [81]. In this study, the dried powders of PP extract were examined for antimicrobial activity against the most common skin and foodborne pathogens. As presented in Table 6, PP microparticles succeeded in inhibiting the growth of all tested microorganisms in relatively low concentrations. The most susceptible were Gram-positive bacteria that can cause skin infections, with a minimum inhibitory concentration (MIC) of 1.75 mg/mL for both *Staphylococcus aureus* and *Staphylococcus epidermidis*. In the study by Costa et al. [82], the antimicrobial activity of polymeric films with incorporated PP extract was likewise effective against *S. aureus* and *S. epidermidis* strains, without a statistical difference between these two species. Also, our results are in accordance with the results presented by Abdollahzadeh et al. [83], where the mentioned microorganisms were sensitive to pomegranate methanolic extract at a slightly higher value of MIC. Among infectious diseases, skin and skin-structure infections are considered the most common ones that lead to significant costs in healthcare systems. Considering the fact that *S. aureus* is found to be the most common pathogen in skin and soft-tissue infections in both ambulatory and hospitalised diabetic patients and that *S. epidermidis* is the most abundant commensal bacterium affecting immunocompromised patients, these results could have a great potential for preparing therapeutic protocols for treating infections related to these bacteria [84,85]. A notable antimicrobial potential of PP powders was also detected against foodborne representatives, *Enterococcus faecalis* (MIC = 2.5–5 mg/mL) and *Shigella flexneri* (MIC = 2.5 mg/mL). Among foodborne illnesses, only shigellosis takes around 700,000 lives annually; therefore, PP extracts could potentially be considered as food preservatives or therapeutic options [86]. *Lysteria monocytogenes,* a foodborne pathogen with the highest fatality rate [87], also showed significant sensitivity to PP extracts with slightly higher MIC values of 5–10 mg/mL. According to the literature, Gram-positive strains were more sensitive to PP extracts compared to Gram-negative [7,81]. The higher sensitivity of Gram-positive bacteria could be related to the mechanism of action of polyphenols based on penetration through cellular walls and hyperacidification of the plasma membrane [24], since for them, this structure is more approachable due to the lack of an outer membrane [88]. It is relevant that the most resistant bacterium in this study was Gram-negative *Pseudomonas aeruginosa*. Along with this bacterial representative, the fungus *Aspergillus brasiliensis* needed higher concentrations of PP extracts for the inhibition of growth. However, even in these cases, the detected minimum bactericidal concentration (MBC) and minimum fungicidal concentration (MFC) values were not higher than doubled MIC. Likewise, for total phenolic content and antioxidant activity, the highest antimicrobial activity was detected for PP microparticles without carrier (A), while B and C showed equal or slightly lower activity. Maroufi et al. [89] also revealed that the antimicrobial activity of PP extracts incorporated in hydrogels was significantly affected by the extracts’ concentration. 

#### 2.3.4. In Silico Molecular Docking Study

**Figure 4:**
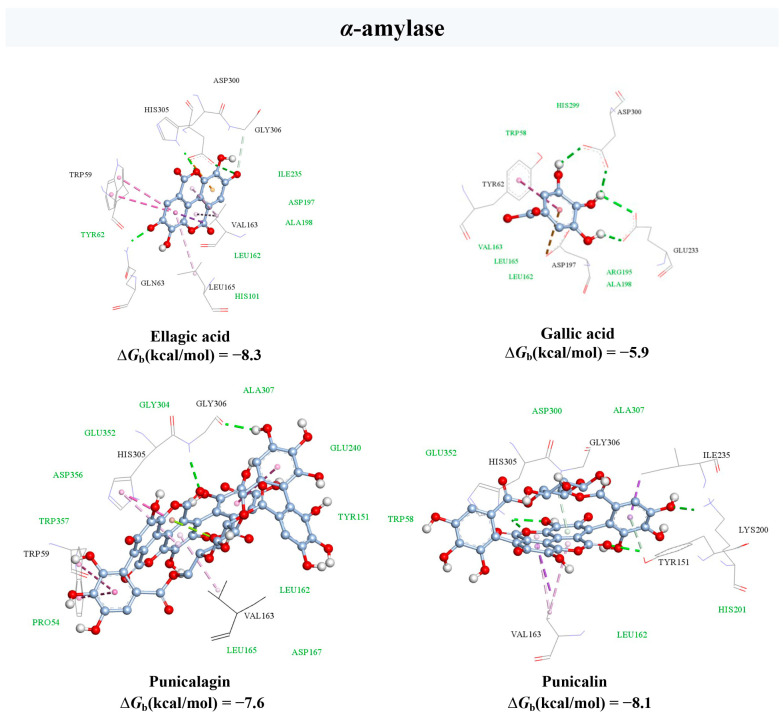
Molecular docking analysis of the main bioactive compounds from pomegranate peel extract and *α*-amylase enzyme. Hydrogen bonds are depicted in green, light pink denotes π–alky interaction, pink refers to π–π, and purple to π–σ interactions. Amino acid residues which form Van der Waal’s interactions are coloured green.

**Figure 5:**
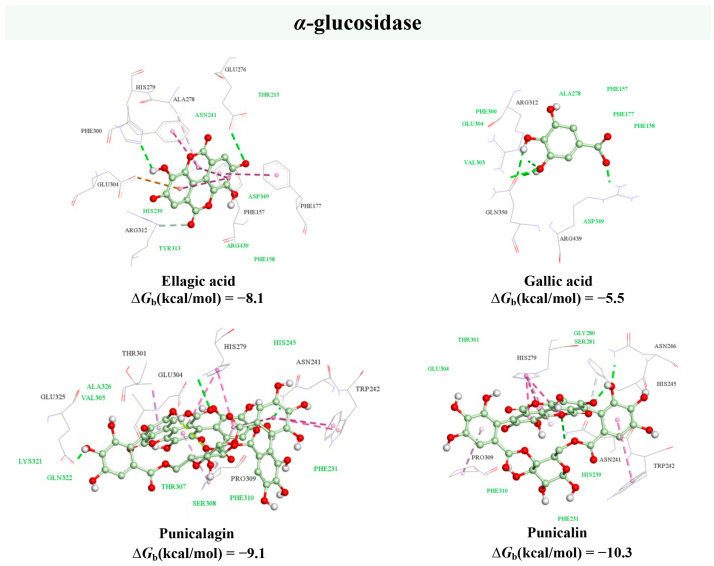
Molecular docking analysis of the main bioactive compounds from pomegranate peel extract and *α*-glucosidase enzyme. Hydrogen bonds are depicted in green, light pink denotes π–alky interaction, pink refers to π–π, and purple to π–σ interactions. Amino acid residues which form Van der Waal’s interactions are coloured green.

The molecular docking method is a widely used in silico structure-based approach for studying protein–ligand interactions at the molecular level. With the aim to support the obtained experimental results, a molecular docking procedure was performed to reveal the binding mechanisms of the main bioactive components of PP extract within the active sites of the *α*-amylase and *α*-glucosidase enzymes (Figure 4 and Figure 5).

According to the AutoDock Vina free energy of binding calculations, ellagic acid was found to have the highest affinity for *α*-amylase enzyme (−8.3 kcal/mol), which confirms the previous findings by Mirab et al. [74]. On the other hand, ellagitannins punicalagin and punicalin were identified as the strongest inhibitors of *α*-glucosidase with binding energies of −9.1 kcal/mol and −10.3 kcal/mol, respectively. These results supported the evidence that *α*-amylase was inhibited by a higher concentration of PP samples compared to *α*-glucosidase. In particular, ellagic acid as the most potent *α*-amylase inhibitor was in a more than 10-fold lower concentration than punicalagin, which was reported as the main *α*-glucosidase inhibitor [90]. 

The docking analysis revealed that the hydrogen bond (HB), van der Waal’s (vdW), and hydrophobic contacts mainly contributed to the interactions between the phenolic compounds of PP extract and the *α*-amylase binding site (Figure 4). Four HBs were found between ellagic acid and the amino acid residues of Gln63, Asp300, His305, and Gly306. The benzene ring of benzopyranone moiety was involved in the formation of hydrophobic π–π, π–σ, and π–alky contacts with the side chains of Trp59, Val163, and Leu165 residues in the active site, respectively. Additional vdW interactions were observed with the side chains of residues, including Tyr62, His101, Leu162, Asp197, Ala198, and Ile235. A similar interaction profile within the active site of *α*-amylase was observed for other studied compounds, as presented in Figure 4. 

The results obtained for the *α*-glucosidase molecular docking study showed that punicalagin forms five conventional and carbon HBs with side chains of Asn241, His279, Glu304, Pro309, and Glu325 within the active region of the target enzyme (Figure 5). Its potency could be also described by the formation of several hydrophobic interactions with Trp242 (π–π), His279 (π–π), Thr301 (π–σ), and Pro309 (π–alky) side-chain residues. Moreover, punicalagin was embedded within the hydrophobic binding pocket of *α*-glucosidase, making vdW interactions with Phe231, His245, Val305, Thr307, Ser308, Phe310, Lys321, Gln322, and Ala326 amino acid residues. Apart from punicalagin, its metabolites, punicalin and ellagic acid, were also found to bind *α*-glucosidase through vdW, hydrophobic, and HB interactions (Figure 5). On the other hand, gallic acid formed only vdW and HB interactions (Figure 5).

The results obtained in the molecular docking analysis are in line with the previously published findings, confirming the accuracy of the performed procedure [90,91]. Altogether, with the experimental hypoglycemic evaluation, it could be concluded that PP microparticles possess promising *α*-glucosidase and *α*-amylase inhibitory activities, providing a basis for their potential use as pharmacological agents for managing type 2 diabetes or the production of functional foods.

### 2.4. Polyphenol Release Kinetics, Diffusion Coefficient, and Diffusion Resistance

**Figure 6:**
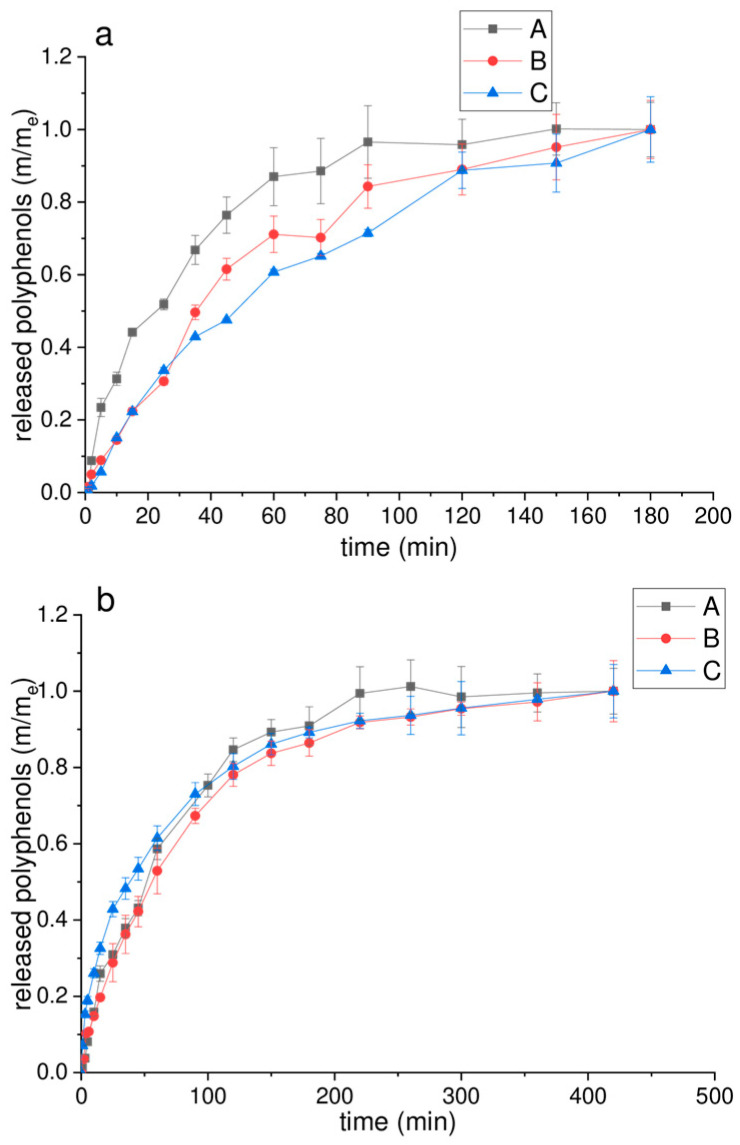
Kinetics of polyphenol release from the obtained spray-dried powders of pure pomegranate peel (PP) extract (A), PP extract with 10% pectin (B), and PP extract with a mixture of 10% pectin and 5% 2-hydroxypropyl-*β*-cyclodextrin (C), observed in Franz diffusion cell in (**a**) simulated gastric fluid (SGF, pH 1.2) and (**b**) simulated intestinal fluid (SIF, pH 6.8) at 37 °C (*m*, the mass of polyphenols at the time of measurement, *m_e_*, the equilibrium mass of polyphenols).

One of the main advantages of microencapsulation includes the formation of preparation with the modified and controlled release of targeted bioactive compounds. Therefore, polyphenol release kinetics from pure PP extract and its’ microencapsulates (with 10% pectin and a mixture of 10% pectin and 5% HP-*β*-CD) in simulated gastrointestinal fluids were investigated employing the Franz diffusion cell. The kinetics of polyphenol release in SGF and SIF are shown in Figure 6a,b, respectively. The results are presented as the dependence of m/m_e_ on time (*m*, the mass of polyphenols at the time of measurement, *m_e_*, the equilibrium mass of polyphenols).

As shown in Figure 6a, the diffusion of polyphenols from pure spray-dried PP extract proceeded rapidly in SGF and reached a plateau after 60 min. On the other hand, the diffusion from pectin and pectin–HP-*β*-CD microencapsulates of PP extract was expectedly slower, and the plateau was reached after 90 and 120 min, respectively (Figure 6a). The presented results showed that the used carriers are able to protect sensitive polyphenol compounds and provide their prolonged release in gastric conditions. Additionally, the results are in agreement with the data obtained in the determination of rehydration (Table 1), where microencapsulates require a longer time for dissolution. The differences in polyphenol recovery between pure extract and microencapsulates can also be explained by a higher specific surface area of small microspheres in spray-dried PP extract (Table 1) (faster release), compared to bigger particles characteristic for spray-dried powders with carriers (slower release) [51]. However, there are differences between the two employed carriers and their ability to deliver polyphenols in an SGF medium. Namely, after the 35th minute, the amount of released polyphenols was higher from the pectin carrier in comparison to a mixture of pectin and HP-*β*-CD (Figure 6a). It can be explained by the formation of pectin gel at pH 1.2 resulting in better release characteristics compared to those at higher pH values [92]. Nevertheless, the release kinetics were different in the SIF medium, and the plateau was reached after 220 min in all tested samples (Figure 6b). Additionally, the release of polyphenols in SIF was higher from the pectin–HP-*β*-CD microencapsulate of PP extract in the first 45 min compared to the pure extract and pectin microencapsulate (Figure 6b). The obtained data showed that a mixture of pectin and HP-*β*-CD did not reduce the recovery of polyphenols in intestinal fluid, where their release and absorption are desirable, preventing their degradation and premature delivery in the acidic pH of gastric conditions. Moreover, the presence of pancreatin and bile salts (in SIF) enhanced polyphenol permeability from microencapsulates. Therefore, the percentage of released polyphenols after 180 min was higher in SIF (in the range from 78% to 90%) than in SGF after the same period (49–54%). 

**Figure 7:**
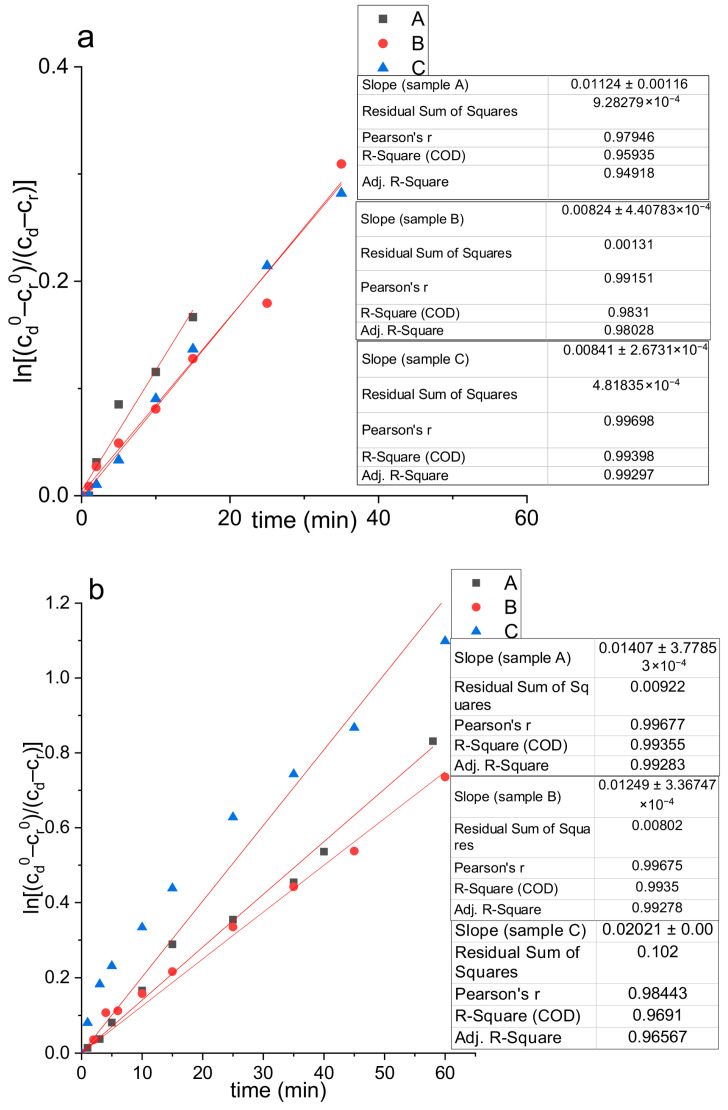
Diffusion of polyphenols from the obtained spray-dried powders of pure pomegranate peel (PP) extract (A), PP extract with 10% pectin (B), and PP extract with a mixture of 10% pectin and 5% 2-hydroxypropyl-*β*-cyclodextrin (C), observed in Franz diffusion cell in (**a**) simulated gastric fluid (SGF, pH 1.2) and (**b**) simulated intestinal fluid (SIF, pH 6.8) at 37 °C approximated by Fick’s second law of diffusion; *c_d_*^0^ and *c_r_*^0^, initial concentration of polyphenols in the donor and receptor part; *c_d_* and *c_r_*, the concentration of polyphenols in the donor and receptor part at the time of measurement.

The polyphenol diffusion from all tested samples approximated by Fick’s second law of diffusion is shown in Figure 7, while the diffusion coefficients (D) and diffusion resistances (R) are presented in Table 7.

According to the results of diffusion coefficients calculated from the slope of the curves from Figure 7a, it can be concluded that, in SGF, the value was higher for pure spray-dried extract (A) in comparison to both types of microencapsulates (B and C) (Table 7). Namely, microencapsulates showed higher resistance to mass transfer (Table 7) and consequently less release of polyphenol compounds than in the case of pure extract, promoting their extended recovery. In the SIF medium, the values of diffusion coefficients calculated from the slope of the curves from Figure 7b followed the trend of pectin–HP-*β*-CD microencapsulate (C) > pure extracts (A) > pectin microencapsulate (B). The release of polyphenol components was faster from the pectin–HP-*β*-CD microencapsulate, i.e., its resistance coefficient was lower compared to the pure spray-dried PP extract (~30%) and pectin microencapsulate (~62%). The obtained data are in agreement with the literature which showed that cyclodextrins can improve the solubility of poorly soluble compounds (also presented in PP extract) due to hydrophilic rims and hydrophobic cavities providing a higher diffusion rate [68,93,94]. On the other hand, the lower release of polyphenols from pectin microencapsulates in SIF can be confirmed by the results of Bermúdez-Oria et al. [95] that have shown the potential binding interactions between pectin and polyphenols. The authors described the formation of stable complexes for the delivery of the phenolic antioxidants to the colon protecting against degradation during intestinal transit.

## 3. Materials and Methods

### 3.1. Plant Material

Pomegranate fruits were collected at the natural locality in a village of Do, Bosnia and Herzegovina, in November 2021. The peel was manually separated from the seeds and subsequently air-dried for 4–6 days at room temperature. A laboratory mill was used to grind the peel, which was further sieved according to the *Yugoslavian Pharmacopeia V* to obtain particles of 0.75 to 2 mm. During the experiment, plant material was stored in paper bags at room temperature. The PP was deposited in the Botanical Garden “Jevremovac”, University of Belgrade (voucher specimen No. BEOU 17742).

### 3.2. Chemicals

Gallic and ellagic acid were purchased from Extrasynthese (Genay Cedex, France), while punicalagin, punicalin, DPPH reagent (2,2-diphenyl-1-picrylhydrazyl), and resazurin were obtained from Sigma Aldrich (St Louis, MO, USA). HPLC-grade acetonitrile was purchased from Merck (Darmstadt, Hesse, Germany) and Folin–Ciocalteu phenol reagent, methanol, sodium carbonate, formic acid, orthophosphoric acid, porcine pancreatic *α*-amylase, and *Saccharomyces cerevisiae α*-glucosidase enzymes were obtained from Sigma Aldrich (St Louis, MO, USA). The potato-starch solution was provided by Thermo Scientific (Waltham, MA, USA). Ethanol (96%) and distilled water were provided by the production site of the Institute for Medicinal Plants Research “Dr. Josif Pančić”. Ultrapure distilled water was generated by a Milli-Q water-purification system (Millipore, Molsheim, France). Hydroxypropyl-*β*-cyclodextrin, vitamin C, acarbose, p-nitrophenyl-α-D-glucopyranoside and 3,5-dinitrosalicylic acid (DNS) were purchased from Acros Organics (Geel, Belgium). Pectin was obtained from CPKelco (Groβenbrode, Germany). Hydrochloric acid, potassium phosphate, sodium hydroxide, pepsin from porcine gastric mucosa, pancreatin from porcine pancreas, and bile salts were from Sigma-Aldrich, USA. Phosphate buffer components (sodium chloride and sodium dihydrogen phosphate, anhydrous) were purchased by Centrohem (Stara Pazova, Serbia). Microbiological culture media Tryptone Soya Broth (TSB), Mueller Hinton Broth (MHB) and Saboraud Dextrose Agar (SDA) were obtained from Himedia (Mumbai, Maharashtra, India).

### 3.3. Extraction Procedure

The liquid extract of PP was obtained using a double percolation method with 50% ethanol (EtOH) as a solvent. The extraction procedure was performed at room temperature with a drug-to-solvent ratio of 1:5. Further, a vacuum evaporator was used to evaporate the obtained extract until the residual EtOH concentration was below 5% in order to prepare the extract for the spray drying method. The extract’s dry weight was determined using a halogen moisture analyser HB43-s (Mettler Toledo, Columbus, OH, USA). The obtained extract was stored in a dark bottle in a cold and dark place until usage. 

### 3.4. Spray Drying Process

The obtained PP liquid extract was further dried using the spray drying technique in the presence and absence of coating materials. The microencapsulation process was performed using 10% (*w*/*w*) pectin, as well as a mixture of 10% (*w*/*w*) pectin and 5% (*w*/*w*) HP-*β*-CD. The concentration of the carriers used in the experiments was based on the calculation of the extract’s dry weight (24.81% *w*/*w*). Initially, carriers were separately dissolved in the extract, while HP-*β*-CD was dissolved 24 h prior to the spray drying process, to enable micellization. Subsequently, the three prepared solutions, pure PP extract, extract with 10% pectin, and extract with a mixture of 10% pectin and 5% HP-*β*-CD, were heated to 40 ℃ with constant magnetic stirring. A LabtexESDTi spray dryer (Labtex, Huddersfield, UK) was used to obtain microparticles of PP extract under the following conditions: 135 ± 5 °C inlet temperature, 70 ± 5 °C outlet temperature, 11 mL/min liquid feed rate, 75 m^3^/h spraying air flow rate, and 2.5 bar atomization pressure, respectively. The obtained powders were stored in glass bottles in desiccators at room temperature prior to further analysis.

### 3.5. Determination of Technological and Physicochemical Properties of the Microparticles

#### 3.5.1. Powder Yield

The PY was calculated using the following Equation (1):

(1) $$PY\left(\%\right)=\frac{m_{p}}{m_{ep}}\times 100$$

where the *m_p_* represents the obtained mass of the spray-dried powder, and *m_ep_* denotes the mathematically calculated expected mass of the powder (sum of the dried residue mass of the collected extract and carrier mass).

#### 3.5.2. Moisture Content

The MC of the prepared PP microparticles was defined thermogravimetrically, at 105 ℃ to a constant mass using a halogen moisture analyser HB43-s (Mettler Toledo, Columbus, OH, USA). The following Equation (2) was used to calculate the moisture content:

(2) $$MC\left(\%\right)=\frac{m_{m}}{m_{s}}\times 100$$

where *m_m_* represents the mass of moisture in the analysed powder. Meanwhile, *m_s_* represents the mass of the sample before the drying process.

#### 3.5.3. Bulk and Tapped Densities, Carr Index, and Hausner Ratio

The previously described method by Vidović et al. [96], with minor modifications, was adopted to determine the bulk density (ρ_bulk_) of the prepared PP powders. Namely, 1 g of the powder was placed in a graduated glass cylinder (5 mL) and subsequently shaken for 5 min (300 rpm). The volume of dried powder was directly measured from the cylinder. The bulk density was calculated as the ratio of the powder mass and the measured volume of powder (g/mL). Similarly, the tapped density (ρ_tapped_) was measured from the cylinder, reading the volume after tapping 120 times (g/mL).

CI and HR were used to describe the flowability and cohesiveness values of the PP powders, respectively. The values were calculated using Equations (3) and (4).

(3) $$CI=\frac{Q_{tapped}-Q_{bulk}}{Q_{tapped}}\times 100$$

(4) $$HR=\frac{Q_{tapped}}{Q_{bulk}}$$

#### 3.5.4. Rehydration and pH

The time needed for the powder to completely rehydrate was evaluated by the addition of 1 g of the PP dried extract into 50 mL of distilled water at room temperature. The mixture was stirred in a glass flask with a magnetic stirrer, and the obtained results were expressed in seconds. Furthermore, the pH value of each sample was determined using a pH meter (Hanna HI 99161, Portugal).

#### 3.5.5. Particle Size Distribution

The particle size distribution of the prepared PP microsized powders was determined by the DLS method, using a Mastersizer 2000 analyser (Malvern Instruments, Worcestershire, UK). Different parameters were examined, including *d*_10_, *d*_50_, and *d*_90_, which denote the sizes of 10%, 50%, and 90% of particles smaller than the remaining particles, respectively. The PDI values represent the indicator of the size distribution width; that calculation is based on the following Equation (5). The surface-weighted mean (D [3,2]), the volume-weighted mean (D [4,3]), and the uniformity of microparticles were also determined.

(5) $$PDI=\frac{d_{90}-d_{10}}{d_{50}}$$

#### 3.5.6. FTIR Spectroscopy Analysis

The structural characterization of the examined PP powders and respective carriers was performed by employing FTIR spectroscopy. The analysis was performed in the range mode from 4000 to 400 cm^−1^ using a resolution of 4 cm^−1^ on a Nicolet iS10 (Thermo Scientific, Stockholm, Sweden) spectrometer in a straight line of examined powder samples. The spectral ranges were measured in duplicate; each sample was analysed independently).

#### 3.5.7. Differential Scanning Calorimetry (DSC)

The thermal characterization of the PP powders was performed using DSC131 Evo (SETARAM Instrumentation, Caluire-et-Cuire, France). The PP samples (5 mg) were placed in aluminium pans (30 µL) and subsequently hermetically sealed, while the empty pan was used as a blind probe. During the heating process, both pans (sample and reference) were initially stabilised at 20 °C for 5 min and further heated to 350 °C, followed by a heating rate of 10 °C/min and a nitrogen flow of 20 mL/min. The empty pans were used to establish a baseline run under the same conditions. Calisto processing 1.38 software equipped with SETARAM instrumentation was used to carry out baseline subtraction and determination of enthalpy values and changes (J/g).

### 3.6. The Content of Total Polyphenols in Microparticles

Prepared microparticles of PP extract were characterised by the content of total phenolic compounds. A previously established spectrophotometric method with Folin–Ciocalteu (FC) reagent was applied with some modifications [97]. Extract samples were diluted (200 µL) and subsequently mixed with a sodium carbonate solution (800 µL) and FC reagent (1000 µL). Samples were incubated for 2 h at room temperature and further quantified by measuring the absorbance at 765 nm against the blank (all reagents except the extract). Experiments were conducted in triplicate, and the results were expressed as a mean value in milligrams of gallic acid equivalents (GAE) per gram of dry weight (mg GAE/g DW).

### 3.7. HPLC Analysis of Individual Phenolic Compounds in Microparticles

The Agilent 1260 RR HPLC instrument (Agilent, Waldbronn, Germany) equipped with a diode-array detector (190–550 nm) was used for chromatographic analysis of the PP samples. The PP microparticles were analysed using a reverse-phase Zorbax SB-C18 (Agilent), analytical column (150 mm × 4.6 mm i.d.; 5 μm particle size), and the mobile phase consisted of 1% (*v*/*v*) solution of orthophosphoric acid in water (A) and acetonitrile (B). According to the following scheme, gradient elution was performed: 0–5 min, 98–90% A; 5–15 min, 90% A; 15–20 min, 90–85% A; 20–25 min, 85–70% A; 25–30 min, 70–40% A; 30–34 min, 40–0% A. Wavelengths for detection were set at 260, 280, 320, 360, and 380 nm, while the flow rate was 1 mL/min. The volume of injection was 3 μL, and the temperature of the column was maintained at 25 °C. Identification of the individual phenolic compounds from PP microparticles was achieved by comparison with the UV spectra and the retention of authentic standard compounds. Calibration curves were used to calculate the amounts of the compounds, and the obtained results were presented as milligrams per gram of dry weight (mg/g DW).

The applied HPLC method was found to be linear within five different concentrations for each of the four analysed compounds. The correlation coefficients (R^2^) for standard gallic acid, ellagic acid, punicalin, and punicalagin were close to 1 (R^2^ > 0.998), indicating a good linear correlation. The limit of detection (LOD) and limit of quantification (LOQ) for the four analysed compounds are presented in Table 8. The values of relative standard deviation (%) were within the 2% limit, indicating that the current method is repeatable.

### 3.8. Biological Evaluation of the Microparticles

#### 3.8.1. Antioxidant Capacity—DPPH Assay

The antioxidant capacity of PP microparticles was assessed by the DPPH assay [98]. An aliquot of 2 mL of sample solution (in 5 different concentrations diluted in methanol) was mixed with 0.5 mL of freshly prepared methanol DPPH solution (0.2 mg/mL). For the blank, 2 mL of methanol were used in place of the sample. The antioxidant capacity was measured spectrophotometrically at 517 nm against the blank (pure methanol) after 30 min of incubation in the dark at room temperature. The results were expressed as the radical-scavenging capacity (RSC) using Equation (6) and further used to calculate the IC_50_ value (the concentration of the extract required to neutralise 50% of free DPPH radicals).

(6) $$RSC(\%)=\left[\frac{\left(A_{c}-A_{s}\right)}{A_{c}}\right]\times 100$$

The *A_S_* refers to the absorbance of the samples at different concentrations, while *A_C_* denotes the absorbance of the control (DPPH solution and methanol). Ascorbic acid was used as a reference standard.

#### 3.8.2. Hypoglycemic Activity

##### α-Amylase Inhibition Assay

The hypoglycemic potential of PP microparticles was evaluated by the method of Ahmed et al. [99] with slight modifications. Solutions of the *α*-amylase enzyme, PP sample, and potato starch (1.0% (*w*/*v*)) were prepared in phosphate buffer (0.1 M, pH 6.9). Sample solutions in serial dilutions were mixed with the *α*-amylase solution and subsequently incubated at 37 °C for 15 min when the starch solution was added. The prepared mixtures were further incubated for 10 min. Lastly, a 3,5-dinitrosalicylic acid solution was added to the mixture, and the samples were kept in a boiling water bath for 15 min with the aim of red colour formation. The control sample was prepared with the phosphate buffer instead of PP samples. The absorbance was measured at 540 nm. The percentage of inhibition was calculated using Equation (7):

(7) $$\alpha -amylase\;inhibition(\%)=\left[\frac{\left(A_{c}-A_{s}\right)}{A_{c}}\right]\times 100$$

where *A_C_* refers to the absorbance of the control, and *A_S_* is the absorbance of the samples. The results were expressed as IC_50_ values representing the concentration of extract required to inhibit 50% of the enzyme in the reaction mixture. The acarbose was used as the reference standard.

##### α-Glucosidase Inhibition Assay

For the evaluation of *α*-glucosidase inhibitory activity of PP microparticles, a slightly modified method of Indrianingsih et al. [100] was applied. Initially, PP spray-dried samples were dissolved in phosphate buffer (0.1 M, pH 6.9) at varying concentrations and incubated with a *p*-nitrophenyl-*α*-D-glucopyranoside solution at 37 °C for 5 min. Afterwards, *α*-glucosidase enzyme solution was added, and the incubation was continued for 15 min when the reaction was stopped by the addition of 0.2 M Na_2_CO_3_ solution. The control sample was prepared with phosphate buffer instead of PP samples, while acarbose was used as the reference standard. The absorbance of the prepared samples was measured at 400 nm, and the percentage of *α*-glucosidase inhibition was calculated using Equation (8):

(8) $$\alpha -glucosidase\;inhibition(\%)=\left[\frac{\left(A_{c}-A_{s}\right)}{A_{c}}\right]\times 100$$

where *A_C_* refers to the absorbance of the control, and *A_S_* is the absorbance of the samples. The results were expressed as IC_50_ values representing the concentration of extract required to inhibit 50% of the enzyme in the reaction mixture.

#### 3.8.3. Antimicrobial Activity

The antimicrobial activity of the PP samples was examined using the broth microdilution method performed according to the recommendations of the National Committee for Clinical Laboratory Standards (CLSI) (2002). The most common microorganisms that can cause foodborne or skin infections were used for the analysis, including *Escherichia coli* O157:H7, *Salmonella enterica* subsp. *enterica* serotype Typhimurium ATCC 14028, *Shigella flexneri* ATCC 12022, *Listeria monocytogenes* ATCC 19114, and *Enterococcus faecalis* ATCC 29212, as recognised causative agents of foodborne infections, and *Escherichia coli* ATCC 8739, *Pseudomonas aeruginosa* ATCC 27853, *Staphylococcus epidermidis* ATCC 12228, *Staphylococcus aureus* ATCC 25923; the fungus *Candida albicans* ATCC 10231, and the mould *Aspergillus brasiliensis* ATCC 16404 as agents of skin infections. The antimicrobial activity was determined as MIC and MBC/MFC.

The antimicrobial assay was performed by the resazurin method in microtiter plates, by detecting the change in colour from purple to pink, as an indicator of microbial growth. Mueller Hinton Broth (MHB) and Tryptic Soy Broth (TSB) were used for bacteria and fungi, respectively. Bacterial strains were adjusted to a final density of 10^6^ CFU/mL, while fungi were adjusted to 2 × 10^4^ CFU/mL. Different concentrations of PP samples were tested for the inhibition of microbial growth, with pure medium and medium with bacteria/fungi without tested powders as negative and positive controls, respectively. Resazurin has been added to all of the microtiter plates’ wells. After inoculation of the plates under normal atmospheric conditions at 37 °C for 24 h for bacteria, and at 25 °C for 3–7 days for fungi, the lowest concentration at which there was no change in the resazurin colour, therefore with no microbial growth, was interpreted as the MIC. Specifically, 2 µL of mediums from wells with MIC and higher tested concentrations were reinoculated in 100 µL of sterile liquid medium and reincubated at 37 °C for 24 h, after which the lowest concentrations without bacterial growth were defined as the MBC. The MFC was determined as the lowest concentration resulting in no growth after reinoculating 10 µL from wells without visible turbidity into Saboraud dextrose agar (SDA) and reincubating at 25 °C for 3 days. All experiments were done in triplicate.

### 3.9. Molecular Docking Analysis

In order to characterise the binding modes of the predominant bioactive compounds from PP extract (gallic acid, ellagic acid, punicalin, and punicalagin) within the active sites of the *α*-glucosidase and *α*-amylase enzymes, molecular docking analysis was performed. Since the 3D structure of *S. cerevisiae α*-glucosidase is not yet revealed, the SWISS-MODEL web server was used to perform the homology modelling analysis [101]. Model generation was based on the structure of isomaltase from the same organism (PDB ID: 3AJ7), which shares a high sequence identity (72.41%) with *α*-glucosidase [102]. The values of the Global Model Quality Estimation (GMQE) and QMEANDisCo Global parameters were 0.95 and 0.91 respectively, which confirmed the credibility of the created homology model. On the other hand, the 3D structure of the porcine pancreatic *α*-amylase enzyme was retrieved from the Protein Data Bank (PDB ID: 1HX0) (https://www.rcsb.org/, accessed on 12 September 2023) [103]. Gallic acid, ellagic acid, punicalin, and punicalagin were downloaded from the PubChem database (https://pubchem.ncbi.nlm.nih.gov/, accessed on 12 September 2023), and structures were energy optimised with an MM2 force field. AutoDock Tools 1.5.7 software was used to define the input files, while AutoDock Vina 1.1.2 was utilised for the docking analysis (exhaustiveness was set to 50 and pose generation was set to 20) [104,105]. BIOVIA Discovery Studio Visualizer v17 was used for the visualization of the obtained binding poses.

### 3.10. In Vitro Release Study

The in vitro release study was performed using a Franz diffusion cell (donation of PermeGear, Inc., Hellertown, PA 18055 USA) with two compartments separated by the acetate–cellulose membrane [51]. The study was conducted for the spray-dried samples, pure PP extract, PP extract with 10% pectin, and PP extract with 10% pectin and 5% HP-*β*-CD in SGF and SIF. SGF contained hydrochloric acid, sodium chloride, and pepsin (pH was adjusted to 1.2 using hydrochloric acid), whereas SIF contained potassium phosphate, sodium hydroxide, pancreatin, and bile salts (pH was adjusted to 6.8 using sodium hydroxide) [106]. The sample was placed in the donor compartment, while the receptor compartment was filled with medium (simulated fluid) and constantly mixed at 360 rpm using magnetic stirring, at 37 °C for simulated fluids, using a water jacket and peristaltic pump. The release of polyphenols was monitored for 4 h (SGF) and 6 h (SIF); the samples were taken from the receptor compartment in certain time intervals. The concentration of polyphenols in the samples was determined spectrophotometrically.

The data of the release study in simulated fluids were used for further calculation of polyphenol diffusion coefficients and diffusion resistances. The diffusion coefficients were determined using Fick’s second law (Equation (9)):

(9) $$D\beta t=\ln\left(\frac{c_{d}^{0}-c_{r}^{0}}{c_{d}-c_{r}}\right)$$

where *D* was the diffusion coefficient, *β* was a geometrical constant, *c_d_* and *c_r_* were concentrations of polyphenols in donor and receptor sections, respectively, at time *t*, whereas *c_d_*^0^ and *c_r_*^0^ were polyphenols concentrations at t = 0. Additionally, diffusion resistance (R) was calculated according to the equation (Equation (10)):

(10) $$R=\frac{\delta }{D}$$

where *δ* is membrane thicknesses.

### 3.11. Statistical Analysis

The data of technological and physicochemical analyses are presented as the mean  ±  standard error. The data was further analysed using a one-way analysis of variances (ANOVA) and Tukey’s HSD *post hoc* test using IBM SPSS 16.0 software. In the case of the results of the antimicrobial assay, a “stricter criteria” rule was applied, common in antimicrobial assays. Namely, the analysis was performed in triplicate and the highest obtained value was taken as MIC and MBC/MFC. Thus, the results are not shown as the average value of several measurements with a standard deviation.

## 4. Conclusions

In recent years, the biowaste obtained from fruit processing has attracted considerable interest in its exploitation as a rich source of bioactive compounds, with potential applications in pharmaceutical, food, and nutraceutical industries. In this study, the spray drying method was employed as an efficient approach for obtaining high-quality microparticles with preserved bioactive compounds of PP extract by using pectin and a mixture of pectin and HP-*β*-CD as coating materials. Spray-dried powders showed a high value of powder yield and satisfied moisture content, flowability, and cohesive properties. The DSC analysis revealed that the carrier addition has a favourable impact on the thermal stability of the examined samples, while additional FTIR analysis suggested that the complexation with carriers occurred on the surface hydrophobic domain. Furthermore, prepared powders with a predominant content of punicalagin, and to a lesser extent punicalin, ellagic and gallic acids, showed considerably high antioxidant activity and a great potential to control elevated glycemic levels. Through in silico molecular docking analysis, it was observed that the ellagic acid possesses the highest affinity to inhibit the *α*-amylase enzyme, while punicalagin and punicalin more selectively inhibited *α*-glucosidase. This study also showed that Gram-positive bacteria, including *S. aureus* and *S. epidermidis*, as representative skin pathogens, were the most susceptible to *P. granatum* peel-extract powders. The results presented herein showed that the used carriers are able to protect sensitive polyphenol compounds and provide their prolonged release in gastric conditions. The obtained results could provide the basis for the development of multifunctional microparticles of PP extract to meet the current market trends in the phytopharmaceutical and healthcare sector as well as the establishment of a feasible method for preserving the physicochemical properties of bioactive compounds from plant material.

## Figures and Tables

**Table 1:** Physical characteristics of the obtained spray-dried powders of pomegranate peel (PP) extract.

Samples	PY (%)	MC (%)	ρ_bulk_ (g/mL)	ρ_tapped_ (g/mL)	CI	HR	pH	Rehydration (s)
A	82.21 ± 3.38 ^a^	2.51 ± 0.15 ^b^	0.32 ± 0.01 ^a^	0.43 ± 0.01 ^a^	26.25 ± 1.26 ^a^	1.35 ± 0.05 ^a^	3.37 ± 0.15 ^a^	21.12 ± 0.75 ^c^
B	78.23 ± 2.56 ^a^	2.43 ± 0.16 ^b^	0.23 ± 0.01 ^b^	0.28 ± 0.01 ^c^	17.89 ± 0.40 ^b^	1.22 ± 0.02 ^b^	3.24 ± 0.06 ^a^	69.85 ± 2.10 ^b^
C	79.95 ± 1.93 ^a^	3.38 ± 0.16 ^a^	0.31 ± 0.01 ^a^	0.36 ± 0.02 ^b^	14.29 ± 0.26 ^c^	1.17 ± 0.05 ^b^	2.81 ± 0.13 ^b^	108.8 ± 3.99 ^a^

A—pure PP extract; B—PP extract with 10% pectin; C—PP extract with a mixture of 10% pectin and 5% 2-hydroxypropyl-*β*-cyclodextrin (HP-*β*-CD); PY—powder yield; MC—moisture content; ρ_bulk_—bulk density; ρ_tapped_—tapped density; CI—Carr index; HR—Hausner ratio; pH—potential of hydrogen. Means followed by different letters differ significantly, based on Tukey’s *post hoc* test at *p* < 0.05, n = 3.

**Table 2:** Particle size (µm), polydispersity index (PDI), surface-weighted mean (D [3,2]), volume-weighted mean (D [4,3]), and uniformity of the obtained spray-dried powders of pomegranate peel (PP) extract.

Samples	d_10_	d_50_	d_90_	PDI	D [4,3]	D [3,2]	Uniformity
A	1.46 ± 0.05 ^c^	5.05 ± 0.24 ^c^	10.06 ± 0.31 ^c^	1.70 ± 0.08 ^b^	5.49 ± 0.15 ^c^	2.64 ± 0.06 ^c^	0.51 ± 0.02 ^c^
B	2.34 ± 0.07 ^a^	8.43 ± 0.41 ^a^	23.56 ± 0.91 ^a^	2.52 ± 0.12 ^a^	13.51 ± 0.37 ^a^	4.00 ± 0.09 ^a^	1.06 ± 0.01 ^a^
C	2.01 ± 0.06 ^b^	7.12 ± 0.32 ^b^	20.58 ± 0.71 ^b^	2.61 ± 0.08 ^a^	9.96 ± 0.44 ^b^	3.61 ± 0.05 ^b^	0.87 ± 0.04 ^b^

A—pure PP extract; B—PP extract with 10% pectin; C—PP extract with a mixture of 10% pectin and 5% 2-hydroxypropyl-*β*-cyclodextrin (HP-*β*-CD); d_10_, d_50_, and d_90_ represent the sizes where 10%, 50%, and 90% of the particles are smaller than the remaining particle in µm, respectively; Means followed by the same letter within the same column are not significantly different according to Tukey’s *post hoc* test at *p* < 0.05, n = 3.

**Table 3:** The transition temperature (T, °C) and enthalpy change (∆H, J/g) of the obtained spray-dried powders of pomegranate peel (PP) extract and used biopolymers.

Samples	T_1_	T_2_	∆H_1_	∆H_2_
A	100.99 ± 7.96 ^a^	142.26 ± 9.18 ^c^	17.88 ± 1.83 ^e^	191.93 ± 18.90 ^a^
B	111.70 ± 10.18 ^a^	150.02 ± 13.95 ^bc^	134.43 ± 11.57 ^c^	145.78 ± 13.89 ^a^
C	109.12 ± 11.53 ^a^	146.89 ± 11.88 ^c^	86.72 ± 7.58 ^d^	77.33 ± 4.17 ^b^
P	112.77 ± 7.66 ^a^	187.59 ± 14.48 ^b^	245.59 ± 21.30 ^a^	45.18 ± 4.13 ^c^
HP-*β*-CD	100.31 ± 11.52 ^a^	328.27 ± 18.77 ^a^	177.93 ± 9.64 ^b^	48.91 ± 3.80 ^bc^

A—pure PP extract; B—PP extract with 10% pectin; C—PP extract with a mixture of 10% pectin and 5% 2-hydroxypropyl-*β*-cyclodextrin (HP-*β*-CD). Means followed by different letters are significantly different according to Tukey’s *post hoc* test al level *p* < 0.05, n = 3. P, pectin; HP-*β*-CD, hydroxypropyl-β-cyclodextrin.

**Table 4:** Content of total phenolic compounds (TPC) and individual compounds (determined in the HPLC analysis) in the obtained spray-dried powders of pomegranate peel (PP) extract.

Sample	TPC (mg GAE/g DW)	Punicalin (mg/g DW)	Gallic Acid (mg/g DW)	Punicalagin (mg/g DW)	Ellagic Acid (mg/g DW)
A	427.88 ± 9.52 ^a^	37.16 ± 4.85 ^a^	5.03 ± 0.63 ^a^	126.82 ± 8.78 ^a^	11.21 ± 1.26 ^a^
B	408.98 ± 14.25 ^a^	34.48 ± 4.24 ^a^	4.41 ± 0.51 ^a^	117.95 ± 10.74 ^a^	10.03 ± 1.28 ^a^
C	373.15 ± 8.38 ^b^	32.72 ± 3.99 ^a^	4.18 ± 0.34 ^a^	112.09 ± 13.63 ^a^	9.62 ± 0.94 ^a^

A—pure PP extract; B—PP extract with 10% pectin; C—PP extract with a mixture of 10% pectin and 5% 2-hydroxypropyl-*β*-cyclodextrin (HP-*β*-CD). Means followed by different letters differ significantly, based on Tukey’s *post hoc* test at *p* < 0.05, n = 3; GAE, gallic acid equivalents; DW, dry weight.

**Table 5:** DPPH radical-scavenging activity and α-amylase and α-glucosidase inhibitory activities of the obtained spray-dried powders of pomegranate peel (PP) extract.

Sample	DPPH IC_50_ (µg/mL)	*α*-Amylase Inhibitory Activity IC_50_ (mg/mL)	*α*-Glucosidase Inhibitory Activity IC_50_ (µg/mL)
A	6.51 ± 0.04 ^c^	7.73 ± 0.68 ^a^	0.25 ± 0.02 ^b^
B	7.92 ± 0.12 ^a^	6.87 ± 0.52 ^a^	0.25 ± 0.03 ^b^
C	7.60 ± 0.04 ^b^	8.21 ± 0.62 ^a^	0.46 ± 0.03 ^a^

A—pure PP extract; B—PP extract with 10% pectin; C—PP extract with a mixture of 10% pectin and 5% 2-hydroxypropyl-*β*-cyclodextrin (HP-*β*-CD). Means followed by different letters differ significantly, based on Tukey’s *post hoc* test at *p* < 0.05, n = 3; IC_50_, the concentration of the sample required to neutralise 50% of DPPH radicals or inhibit 50% of the enzyme in the reaction mixture.

**Table 6:** Antimicrobial activity of the obtained spray-dried powders of pomegranate peel (PP) extract on most common foodborne and skin pathogens.

		Sample					
		A		B		C	
Microorganisms		MIC (mg/mL)	MBC (mg/mL)	MIC (mg/mL)	MBC (mg/mL)	MIC (mg/mL)	MBC (mg/mL)
**skin**	*E. faecalis*	2.5	5	5	5	5	5
	*L. monocytogenes*	5	10	10	10	10	10
	*E. coli*	10	10	10	10	10	10
	*S. Typhimurium*	10	10	10	10	10	10
	*S. flexneri*	2.5	5	2.5	5	2.5	5
**foodborne**	*S. aureus*	1.75	2.5	1.75	2.5	1.75	2.5
	*S. epidermidis*	1.75	2.5	1.75	2.5	1.75	2.5
	*E. coli*	2.5	5	5	5	2.5	5
	*P. aeruginosa*	10	17.5	15	20	15	17.5
	*C. albicans*	5	7.5	10	15	10	12.5
	*A. brasiliensis*	10	20	20	30	15	20

A—pure PP extract; B—PP extract with 10% pectin (B); C—PP extract with a mixture of 10% pectin and 5% 2-hydroxypropyl-*β*-cyclodextrin (HP-*β*-CD) (C); MIC—minimal inhibitory concentration; MBC/MFC—minimal bactericidal/fungicidal concentration; “stricter criteria” rule was applied, common in antimicrobial assays (n = 3 and the highest obtained value was taken as MIC and MBC/MFC). Tested skin microorganisms: *Enterococcus faecalis*, *Lysteria monocytogenes*, *Escherichia coli*, *Salmonella enterica* serotype Typhimurium, and *Shigella flexneri*. Tested foodborne microorganisms: *Staphylococcus aureus*, *Staphylococcus epidermidis*, *Escherichia coli*, *Pseudomonas aeruginosa*, *Candida albicans,* and *Aspergillus brasiliensis*.

**Table 7:** Diffusion coefficients (D) and diffusion resistances (R) of the obtained spray-dried powders of pomegranate peel (PP) extract observed in Franz diffusion cell in simulated gastric fluid (SGF, pH 1.2) and simulated intestinal fluid (SIF, pH 6.8) at 37 °C.

Medium	Sample	D (m^2^/s)	R (s/m)
	A	7.52 × 10^−9^	4.41 × 10^5^
SGF	B	5.52 × 10^−9^	7.38 × 10^5^
	C	5.63 × 10^−9^	7.23 × 10^5^
	A	9.42 × 10^−9^	4.32 × 10^5^
SIF	B	8.36 × 10^−9^	7.87 × 10^5^
	C	1.35 × 10^−8^	3.01 × 10^5^

A—pure PP extract; B—PP extract with 10% pectin; C—PP extract with a mixture of 10% pectin and 5% 2-hydroxypropyl-*β*-cyclodextrin.

**Table 8:** Limit of detection (LOD) and limit of quantification (LOQ) for analysed compounds.

Parameter	Gallic Acid	Ellagic Acid	Punicalin	Punicalagin
LOD (μg/mL)	6.25	12.50	8.25	12.50
LOQ (μg/mL)	18.50	30.20	20.55	20.50
Linear range (μg/mL)	50–800	35–560	50–800	50–800
R^2^	0.9995	0.9998	0.9996	0.9998

R^2^—The correlation coefficient.

## Data Availability

The data are contained within the manuscript.
